# Multifocal calcifying fibrous tumor at six sites in one patient: a case report

**DOI:** 10.1186/1477-7819-12-235

**Published:** 2014-07-29

**Authors:** Faisal Azam, Madhuchanda Chatterjee, Sheila Kelly, Maria Pinto, Amey Aurangabadkar, M Farooq Latif, Ernie Marshall

**Affiliations:** 1 Department of Medical Oncology, North Wales Cancer Treatment Centre, Glan Clwyd Hospital, Rhyl LL18 5UJ, United Kingdom; 2 Department of Medical Oncology, Clatterbridge Cancer Centre, Clatterbridge Road, Bebington, Wirral CH63 4JY, United Kingdom; 3 Department of Histopathology and Radiology, Whiston Hospital, warrington Road, Prescot, Merseyside L35 5DR, United Kingdom

## Abstract

Calcifying fibrous tumors (CFT) are rare benign tumors. They usually affect children and young adults and the incidence is equal in males and females. The usual clinical presentation is that of a painless mass. A computed tomography scan typically reveals a well-demarcated calcified lesion. CFT usually presents as a solitary mass and the commonest sites of occurrence are in soft tissues, the pleura, or the peritoneum. Multifocal occurrences at the same site have also been reported. The first case of CFT was reported in 1988. We present a rare case of multiple calcifying fibrous tumors at multiple sites in the same patient. To the best of our knowledge, this is the first ever reported case of multifocal CFT atsix different anatomical sites in one patient.

## Background

Calcifying fibrous tumors (CFT) are rare benign tumors. They usually affect children and young adults and the incidence is equal in males and females. The usual clinical presentation is that of a painless mass. A computed tomography scan typically reveals a well-demarcated calcified lesion. CFT usually presents as a solitary mass and the commonest sites of occurrence are in soft tissues, the pleura, or the peritoneum. Multifocal occurrences at the same site have also been reported. The first case of CFT was reported in 1988 [1]. We present a rare case of multiple calcifying fibrous tumors at multiple sites in the same patient. To the best of our knowledge, this is the first ever reported case of multifocal CFT at six different anatomical sites (the paraspinal, pelvis, lungs, spleen, right adrenal gland and liver) in one patient.

## Case presentation

**Figure 1:**
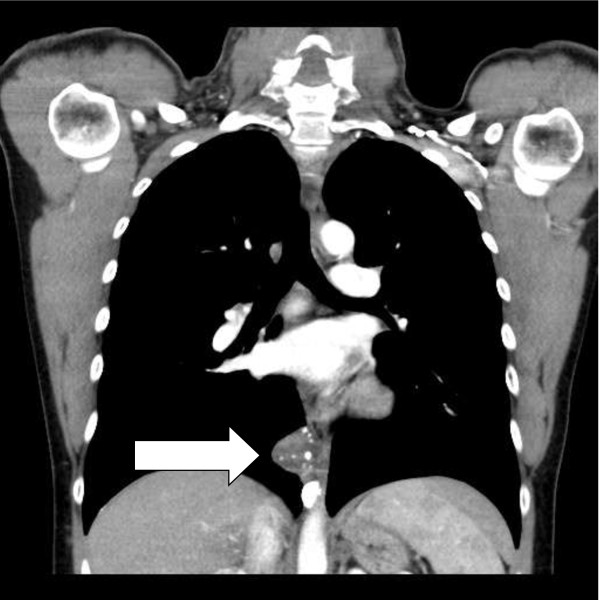
CT image of CFT in the paraspinal region (arrow).

**Figure 2:**
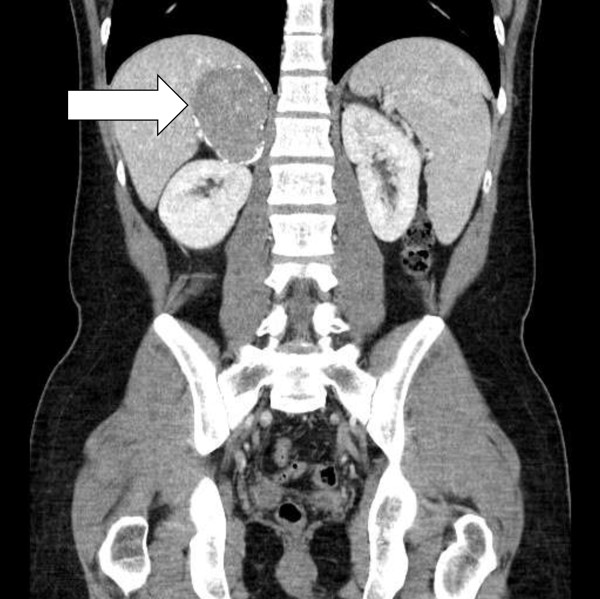
CT image right adrenal gland CFT.

**Figure 3:**
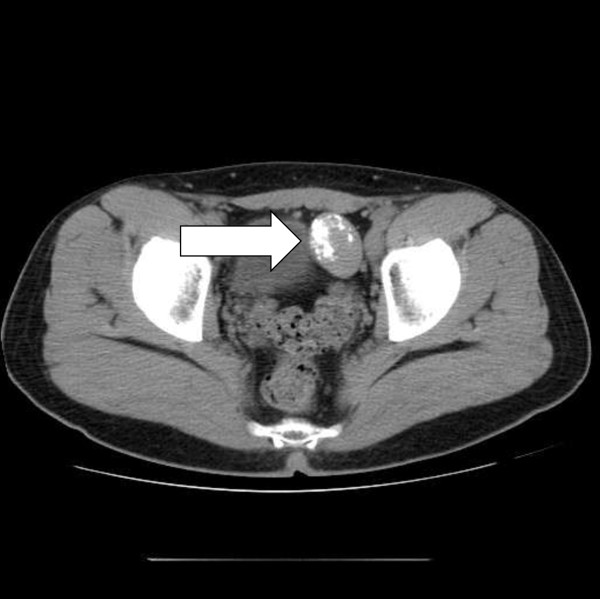
CT image of CFT in the pelvis.

**Figure 4:**
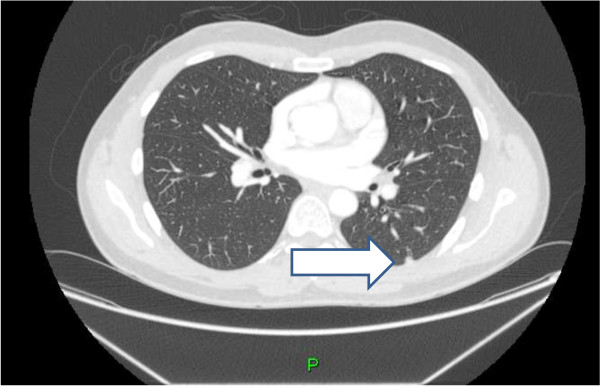
CT image of a lung nodule.

**Figure 5:**
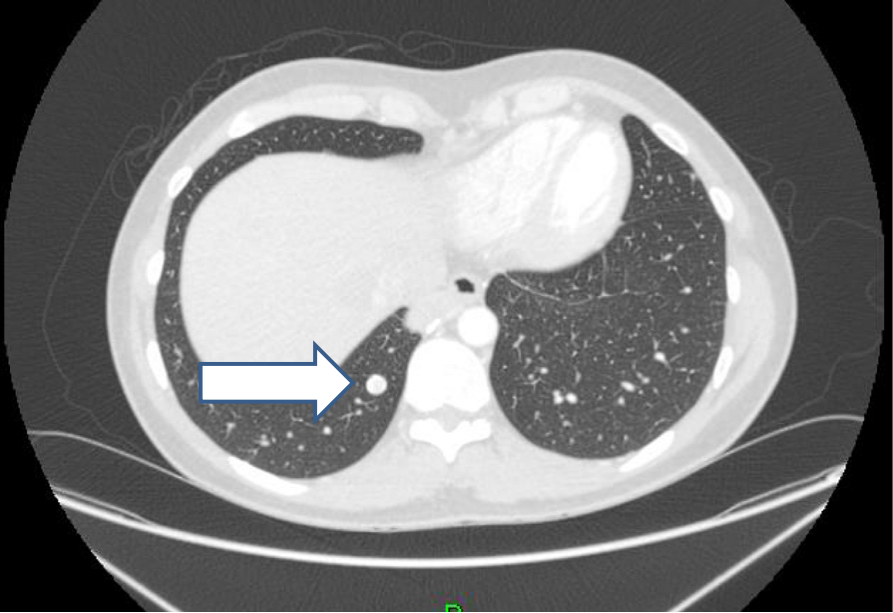
CT image of a lung nodule.

A 31-year-old caucasian man presented with a three-week history of abdominal pain on urination and altered bowel habits. Apart from tenderness and fullness in the right loin, his clinical examination was unremarkable. There were no clinical signs of autoimmune disease. He was physically fit, with no history of major illness. The results of his renal function, liver enzyme, bone profile, thyroid function, and full blood count tests, as well as an ultrasound of his kidneys, were normal. A computed tomography (CT) scan of his chest, abdomen, and pelvis reported multiple calcified masses in his right adrenal gland, right paraspinal region, left pelvis, spleen, liver, and lung nodules (Figures 1, 2, 3, 4 and 5).

He was initially investigated for metastatic adrenal cortical carcinoma, pheochromocytoma, or metastatic sarcoma due to the right adrenal mass found in his CT scan. His random serum cortisol levels were raised and his 24-hour urinary noradrenalin, adrenalin, and dopamine levels were normal. Serum tumor markers such as carcinoembryonic antigen (CEA), alpha fetoprotein (AFP), and beta human chorionic gonadotropin (β-HCG) were normal. An initial CT-guided biopsy from the pelvic mass showed only unremarkable fibrofatty tissue and was hence concluded as non-diagnostic (Additional file 1).

We performed an open biopsy of the pelvic mass and the histopathology confirmed the mass as dense calcified fibrous tissue with a possiblehyalinized leiomyoma or fibroma, with no evidence of malignancy. He had a repeat CT scan after three months which showed no change in multiple masses. A laparotomy was performed because of ongoing symptoms of abdominal pain. A 5-cm retroperitoneal mass was excised from the lateral pelvic wall. This was described as a firm nodule with a smooth surface and a slightly whorled appearance on the cut surface. Microscopically it consisted of well-circumscribed tumor consisting of collagen with scattered plump fibroblasts, a mild diffuse lymphocytic, and plasma cell infiltrate with occasional lymphoid follicles. Neither necrosis nor mitosis was identified. Inflammatory myofibroblastic tumor (IMT) was considered as a possible diagnosis but CFT was considered to be more likely. Both these resection specimens were sent for a second opinion by a soft tissue pathologist who confirmed the diagnosis of CFT as there was no expression of anaplastic lymphoma kinase 1 (ALK1). The patient’s symptoms of pelvic pain resolved after surgery. His case was discussed in a specialist multidisciplinary meeting and it was decided not to resect other masses as he was currently asymptomatic. A multidisciplinary team meeting decided against biopsying other lesions as they were not showing any signs of change and were radiologically similar to the one resected. He had surveillance CT scans every four months and there was no change in the remaining tumors. He remained very well and asymptomatic more than two years after his diagnosis of CFT. He is currently continuing with six-monthly clinical follow-ups. He remains well and asymptomatic.

## Discussion

CFT is a rare benign tumor presenting as a solitary calcified mass or multiple masses in one particular organ. The pathogenesis is unknown. The mass or masses are characterized as a collection of dense hyalinized collagenous tissue interspersed with benign-appearing spindle cells, psammomatous or dystrophic calcifications with lymphoplasmacytic inflammatory infiltrate [1]. It was originally described by Rosenthal and Abdul-Karim as a childhood fibrous tumor with psammoma bodies [2]. Fetsch *et al*. reported 10 cases of CFT in 1993 and named this tumor’calcifying fibrous pseudotumor’ for the first time [1].

The most common site of CFT reported in the literature is the pleura [3,4]. On immunohistochemical staining these tumors are usually negative for CD34 (vascular marker) and cytokeratin (epithelial marker) [5]. The fibroblasts stain positive for vimentin (mesenchymal cell marker) and negative for cytokeratins and CD34. Some investigators have proposed that CFT is a late sclerosing stage of inflammatory myofibroblastic tumor_._ Inflammatory myofibroblastic tumor is, however, distinguished from CFT by being generally more cellular; less hyalinized and typically lacking in calcification. Unlike IMT, CFT rarely expresses ALK by immunohistochemistry, suggesting that CFT is a different clinicopathologic entity than IMT. CFT can be asymptomatic for many years before presenting with symptoms. Symptoms vary with the site of occurrence and common presentations are those of pressure symptoms. The treatment for CFT is as it was in our case - surgical resection with long-term follow-up. Recurrence is extremely rare [5,6].

On a review of the literature we found 103 cases of CFT. Of these, a total of 41 patients were male and 62 patients were female. Eighty patients had solitary tumors and 23 patients had multiple lesions. All of the patients who had multifocal CFT were limited to one or two anatomical sites [4,7-10]. To the best of our knowledge, our case report is the first case with multifocal CFT at six different sites in one patient.

## Conclusions

CFT is a rare benign tumor with a good prognosis after resection. It can be misdiagnosed on imaging as metastatic carcinoma. Histological confirmation by an experienced pathologist can confirm the diagnosis. Recurrence after complete resection has not been reported.

## Consent

Written informed consent was obtained from the patient for publication of this case report and any accompanying images. A copy of the written consent is available for review by the editor in chief of this journal.

## Competing interests

The authors declare that they have no competing interests.

## Authors’ contribution

FA, Manuscript writing and proof reading. MC, Manuscript writing and proof reading. SK, Providing pathology specimen/slides and proof reading. MP, Providing pathology specimen/slides and proof reading. AA Proof reading. MFL, Manuscript writing and proof reading. EM Proof reading. All authors have read and approved the final version of the manuscript.

## Supplementary Material

Additional file 1 Histological appearance of pelvic CFT. Click here for file
