# Supplementary figures and images for: Multifocal calcifying fibrous tumor at six sites in one patient: a case report
Source: World J Surg Oncol. 2014 Jul 29;12:235. doi: 10.1186/1477-7819-12-235 (PMC4127171; doi:10.1186/1477-7819-12-235)

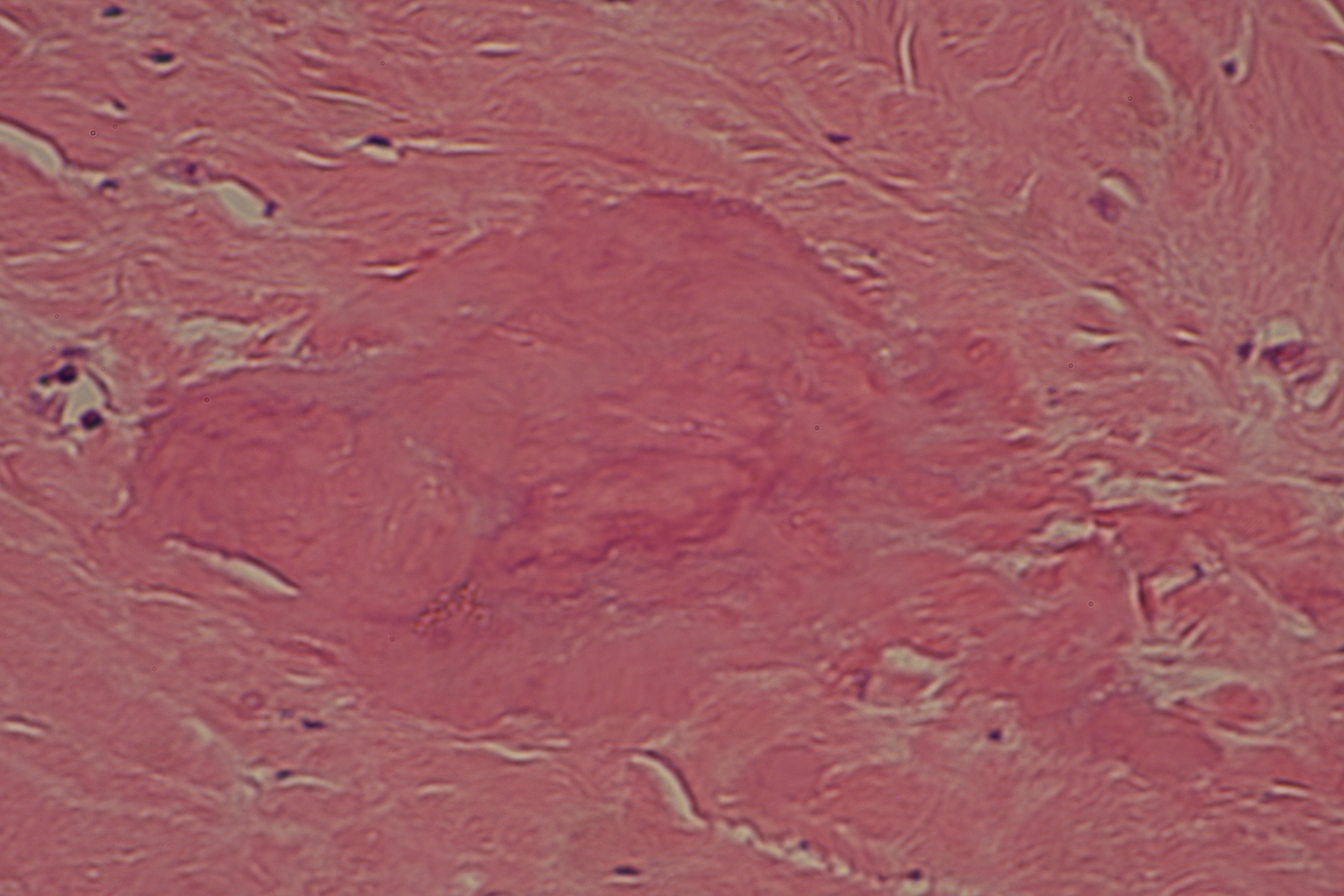

Supplement: Additional file 1 — Histological appearance of pelvic CFT. [file 1477-7819-12-235-S1.jpeg]
